# Increased Frequency of Tim-3 Expressing T Cells Is Associated with Symptomatic West Nile Virus Infection

**DOI:** 10.1371/journal.pone.0092134

**Published:** 2014-03-18

**Authors:** Marion C. Lanteri, Michael S. Diamond, Jacqueline P. Law, Glen M. Chew, Shiquan Wu, Heather C. Inglis, Derek Wong, Michael P. Busch, Philip J. Norris, Lishomwa C. Ndhlovu

**Affiliations:** 1 Blood Systems Research Institute, University of California at San Francisco, San Francisco, California, United States of America; 2 Departments of Medicine, Molecular Microbiology, and Pathology & Immunology, Washington University School of Medicine, St. Louis, Missouri, United States of America; 3 Department of Tropical Medicine, John A. Burns School of Medicine, University of Hawaii, Honolulu, Hawaii, United States of America; 4 Department of Laboratory Medicine, University of California San Francisco, San Francisco, California, United States of America; 5 Department of Medicine, University of California San Francisco, San Francisco, California, United States of America; Washington University, United States of America

## Abstract

More than a decade after West Nile virus (WNV) entered North America, and despite a significant increase in reported cases during the 2012 and 2013 seasons, no treatment or vaccine for humans is available. Although antiviral T cells contribute to the control of WNV, little is known about their regulation during acute infection. We analyzed the expression of Tim-3 and PD-1, two recently identified T cell negative immune checkpoint receptors, over the course of WNV infection. Symptomatic WNV+ donors exhibited higher frequencies of Tim-3^+^ cells than asymptomatic subjects within naïve/early differentiated CD28^+/–^CD57^–^CD4^+^ and differentiated CD28^–^CD57^–^CD8^+^ T cells. Our study links Tim-3-expression on T cells during acute WNV infection with the development of symptomatic disease, suggesting Tim-3 and its ligands could be targeted therapeutically to alter anti-WNV immunity and improve disease outcome.

## Introduction

Over the past 13 years West Nile virus (WNV) has been responsible in the United States (US) alone for more than 38,000 documented infections, of which 16,453 developed neuroinvasive disease, and 1,579 died [1]. Based on serological analysis, it is projected that over 3 million people were infected with WNV in the US from 1999 to 2010 [2]. With no specific treatment or vaccine licensed against WNV for use in humans, an improved understanding of host-virus interface and novel approaches to therapy are needed.

Increased age [3] and host genetic background [4], [5], [6] have been associated with symptomatic disease or failure to control WNV infection. Studies in rodents and humans have established the importance of innate and adaptive immunity in the control and clearance of WNV infection and prevention of its complications [7]. Immunocompromised humans and animals develop more severe symptoms and disease after WNV infection [8]. The humoral immune response is important in the control of WNV viremia and prevention of spread to the central nervous system, and antiviral T cells function to clear WNV from infected tissues and limit the extent of WNV disease in the central nervous system (CNS) [7].

Although WNV-specific CD8^+^ T cell responses are required to clear WNV from the CNS, these responses can contribute to immunopathology, which is characterized by excessive neuronal injury and inflammation [9], [10]. Although research has begun to clarify the relationship between immune protection and disease, the precise correlates of protective T cell immunity in humans remain uncharacterized. Determining the nature of protective rather than pathogenic T cell responses and identifying strategies to modulate such activities could reduce the risks of neurological complications or death in recently infected persons, as well as inform vaccine strategies that optimize cell-mediated immune responses**.**

Several counter-regulatory mechanisms have been suggested to control the functional fate of T cells. While negative regulatory mechanisms limit host tissue damage by dampening inflammation, this could have adverse effects by suppressing requisite antiviral effector T cell responses [11]. In the course of chronic antigen persistence during viral infection, T cells advance through sequential stages of ‘exhaustion’ that are characterized by expression of multiple inhibitory receptors, including the programmed death-receptor 1 (PD-1) [12], which is associated with T cells retaining proliferative but losing cytokine production capacities, and the T cell immunoglobulin and mucin domain 3 receptor (Tim-3), which is linked to poor proliferative and polyfunctional cytokine production capacities in T cells [12]. Higher expression of Tim-3 on PD-1 expressing T cells correlates with clinical progression in multiple chronic viral infections [13]. However, the role of inhibitory T cell receptor checkpoints in acute viral infections such as WNV remains less well characterized.

The present study investigated the dynamics of Tim-3 and PD-1 inhibitory receptor expression through the evolution of acute WNV infection. Our results reveal an association between increased frequencies of Tim-3^+^ T cells and the development of symptomatic WNV disease in humans, suggesting that Tim-3 and its ligands could be targeted therapeutically to limit the development of symptomatic WNV disease.

## Materials and Methods

### Human subjects

Thirty-two WNV-infected subjects were enrolled in 2007 by the Blood Systems Research Institute (BSRI, San Francisco, CA) from donors in United Blood Services blood centers throughout the US. Blood donors whose donation tested positive for WNV RNA (WNV+) with the WNV Procleix transcription mediated amplification assay (Novartis Diagnostics) were enrolled in follow-up studies after obtaining written informed consent. Questionnaires were administered to determine symptoms during the three week period around initial blood donation (index). Samples were collected at regional blood centers during follow-up visits and shipped to BSRI. Based on symptom data, WNV+ blood donors were classified as asymptomatic (n = 24) or symptomatic (n = 8) as previously described [14]. The control subjects in this study included 16 apheresis blood donors who tested negative for WNV RNA (WNV−) (no demographic data available) and 10 healthy controls who had provided written consent and were negative for WNV IgM. The research involving human participants reported in this study was approved by the UCSF Committee on Human Research (IRB # 10-01255 and # 11-06262).

### Peripheral blood mononuclear cell isolation

The peripheral blood mononuclear cells (PBMCs) and plasma used in this study were derived from blood drawn from 32 WNV+ blood donors at regional blood centers. Samples were processed within 24 hours, with PBMCs and plasma cryopreserved for future use. The samples used in this study were collected a mean of 14 days, 1 month, 3 months, and 12 months post-index donation. The PBMCs from the 16 WNV- uninfected donors were derived from discarded leukoreduction chambers collected on apheresis machines after platelet donations and were cryopreserved in liquid nitrogen [15].

### Human flow cytometry analysis

Cryopreserved human PBMCs were thawed and stained with the LIVE/DEAD® aqua amine-reactive dye (Invitrogen). The cells were washed and surface-stained with a cocktail of antibodies consisting of Energy Coupled Dye (ECD)-conjugated anti-CD3 (clone UCHT1, Beckman Coulter), Alexa Fluor® 700 (A700)-conjugated anti-CD4 (clone RPA-T4, BD Pharmingen), APC-Cyano dye 7 (APC-Cy7)-conjugated anti-CD8 (clone SK1, BD Pharmingen), Phycoerythrin-Cyano dye 7 (PE-Cy7)-conjugated anti-CD28 (clone CD28.2, eBioscience), Fluorescein isothiocyanate (FITC)-conjugated anti-CD57 (clone HCD57, BioLegend), Phycoerythrin (PE)-conjugated anti-Tim-3 (clone 344823, R&D Systems, Inc.), and Allophycocyanin (APC)-conjugated anti-PD-1 (clone EH12.2H7, BioLegend) antibodies. Cells were then washed and fixed with BD Stabilizing Fixative buffer (BD Bioscience). In separate experiments cells were stained with the LIVE/DEAD® aqua amine-reactive dye and surface-stained with PE-conjugated HLA-A02-restricted SVG9 (E peptide) tetramers [16] (prepared by the NIH Tetramer Facility, Atlanta, GA) for 45 minutes at 37°C before being washed and surface-stained with a cocktail of antibodies consisting of ECD-conjugated anti-CD3 (clone UCHT1, Beckman Coulter), APC-Cy7-conjugated anti-CD8 (clone SK1, BD Pharmingen), and A700-conjugated anti-Tim-3 (Clone 344823, R&D Systems, Inc.) monoclonal antibodies (mAbs) before final wash and fixation in BD Stabilizing Fixative buffer (BD Bioscience). In separate experiments cells were incubated for 2 hours before the addition of Brefeldin A (5 μg/mL) for overnight incubation in unstimulated and stimulated conditions with anti-CD3 plus anti-CD28 mAbs (BD Pharmingen), with a pool of 8 WNV peptides corresponding to immunodominant CD8^+^ T cell epitopes in membrane, envelope, and nonstructural 3 and 4B proteins identified in WNV+ subjects [17] (using 10 μg/mL of individual peptides) along with the HLA-A02-restricted SVG9 peptide (at a 1/200 dilution from a 1.2 mg/mL stock solution in a final volume of 100 μL of RPMI-1640 with 10% FBS). Cells were stained with the LIVE/DEAD® aqua amine-reactive dye, washed and surface stained with FITC-conjugated anti-CD4 (clone OKT4, BioLegend), APC-Cy7-conjugated anti-CD8 (clone SK1, BD Bioscience), and BV421-conjugated anti-Tim-3 (Clone F38-2E2, BioLegend) mAbs. After washing, cells were fixed and permeabilized with Caltag mediums A and B (Invitrogen) before addition of ECD-conjugated anti-CD3 (clone UCHT1, Beckman Coulter) and APC-conjugated IFN-γ (clone B27, BioLegend) mAbs. Then cells were washed and fixed in 1% paraformaldehyde. The cells used for FMO conditions were stained and acquired in parallel. The acquisition was performed using an LSRII instrument (BD Biosciences). At least 100,000 events were collected and analyzed with the FlowJo software (TreeStar).

### Statistical analysis

GraphPad Prism and R statistical software was used for data analysis. A two-tailed *t* test was used where appropriate. For data with non-Gaussian distributions (i.e., cell marker frequency measurements), the non-parametric Mann-Whitney rank sum test for two independent populations was performed. The false discovery rates (FDR) were derived from the Benjamini and Hochberg procedure. While accounting for data dependency, the generalized estimating equations (GEE) method was used to compare the panels of multiple longitudinal measurements. Statistical significance was considered for *p*-values <0.05 and FDRs≤0.1.

## Results

### Symptomatic WNV+ subjects have higher frequencies of Tim-3-expressing CD4^+^ and CD8^+^ T cells than asymptomatic WNV+ subjects

**Figure 1:**
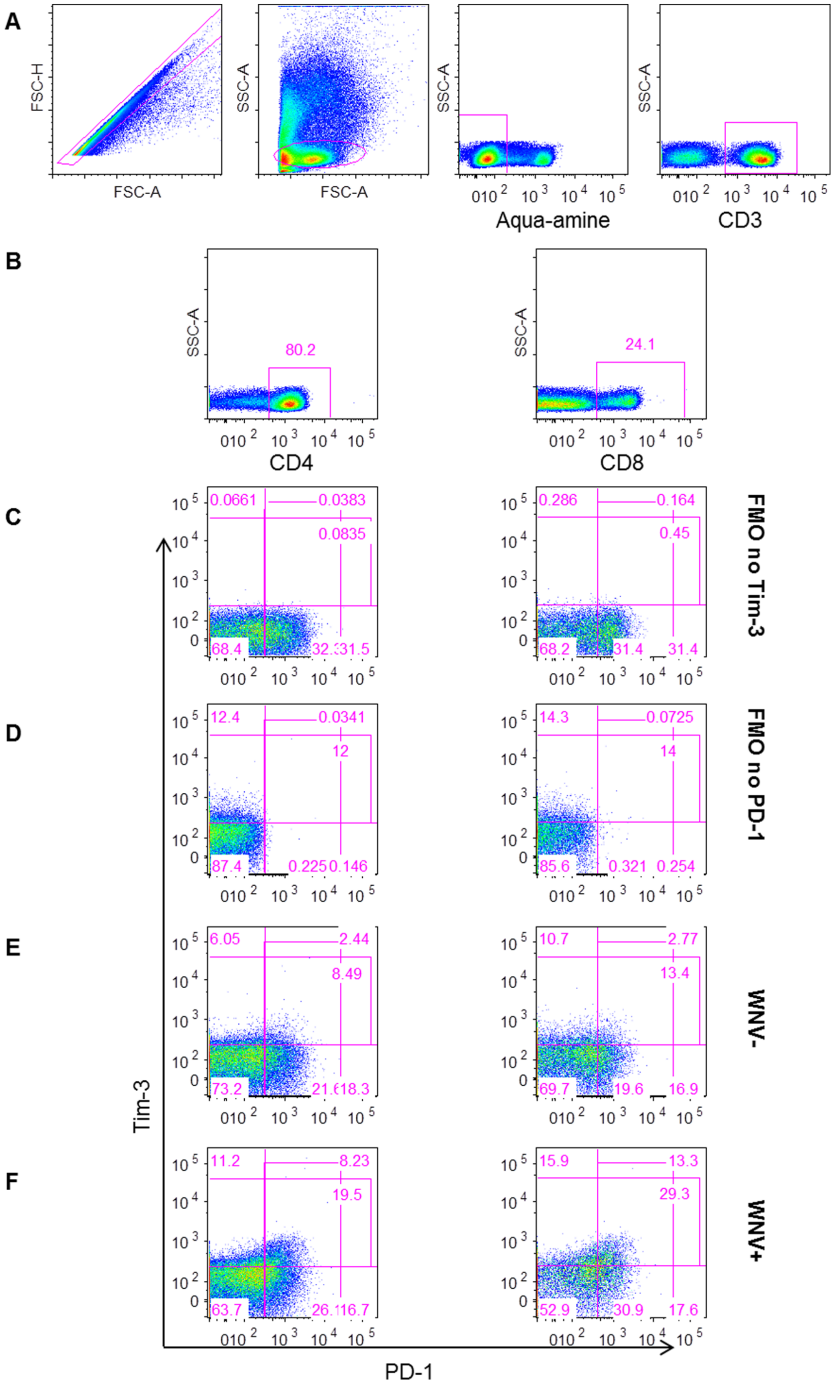
Gating strategy for measuring Tim-3 and PD-1 inhibitory receptors on T cells. The plots show (A) the gating strategy for live CD3^+^ lymphocytes, (B) for CD4^+^ (left) and CD8^+^ (right) T cells. Gates were set on FMO no Tim-3 (C) and FMO no PD-1 (D). The gating of cells expressing Tim-3 and PD-1 is shown for representative (E) WNV− and (F) WNV+ subjects day 14 post-index donation.

We characterized the cell surface expression of Tim-3 and PD-1 on T cells over the course of acute WNV infection in a cohort of WNV-positive (WNV+) human blood donors followed from the earliest stage of viremia (**Table 1**) throughout convalescence [18]. The proportion of Tim-3 and PD-1 expressing CD4^+^ and CD8^+^ T cells was measured by flow cytometry (**Figure 1**) from PBMCs collected at days 14, 30, 90, and 365 after blood donation (post-index) from 32 WNV+ blood donors, and was compared to cells collected at a single time-point from 26 WNV-negative normal controls (WNV−).

**Table 1:** WNV+ subject characteristics.

Subject	Symptom number				Gender^b^	Age	IgM^c^	IgG^c^	Donor Status^d^
	Before donation	Index donation	After donation	Peak symptom number^a^					
**267**	**0**	**0**	**0**	**0**	**F**	**52**	**-**	**-**	AS
**247**	**1**	**0**	**N/A**	**1**	**F**	**29**	**-**	**-**	AS
**257**	**0**	**0**	**1**	**1**	**M**	**58**	**-**	**-**	AS
**437**	**1**	**0**	**0**	**1**	**F**	**57**	**-**	**-**	AS
**227**	**1**	**0**	**1**	**1**	**F**	**69**	**-**	**-**	AS
**17**	**2**	**2**	**0**	**2**	**M**	**52**	**-**	**-**	AS
**207**	**1**	**1**	**2**	**2**	**F**	**57**	**-**	**-**	AS
**357**	**0**	**0**	**2**	**2**	**F**	**46**	**-**	**-**	AS
**387**	**2**	**0**	**0**	**2**	**F**	**18**	**-**	**-**	AS
**137**	**2**	**0**	**N/A**	**2**	**M**	**63**	**-**	**-**	AS
**407**	**0**	**1**	**2**	**2**	**F**	**65**	**-**	**-**	AS
**127**	**3**	**0**	**0**	**3**	**M**	**26**	**-**	**-**	AS
**327**	**3**	**0**	**0**	**3**	**M**	**58**	**-**	**-**	AS
**77**	**0**	**0**	**0**	**0**	**M**	**77**	**+**	**-**	AS
**497**	**0**	**0**	**0**	**0**	**F**	**69**	**+**	**-**	AS
**517**	**0**	**0**	**N/A**	**0**	**F**	**65**	**+**	**-**	AS
**487**	**2**	**0**	**0**	**2**	**F**	**65**	**+**	**-**	AS
**177**	**0**	**0**	**0**	**0**	**M**	**60**	**+**	**+**	AS
**367**	**0**	**0**	**0**	**0**	**M**	**36**	**+**	**+**	AS
**187**	**1**	**0**	**1**	**1**	**M**	**37**	**+**	**+**	AS
**297**	**2**	**0**	**0**	**2**	**F**	**61**	**+**	**+**	AS
**307**	**0**	**0**	**0**	**0**	**M**	**38**	**+**	**E**	AS
**627**	**2**	**1**	**0**	**2**	**F**	**60**	**+**	**E**	AS
**57**	**0**	**1**	**0**	**1**	**M**	**69**	**E**	**+**	AS
**87**	**1**	**0**	**4**	**4**	**F**	**59**	**-**	**-**	S
**477**	**0**	**0**	**4**	**4**	**M**	**65**	**-**	**-**	S
**47**	**0**	**0**	**5**	**5**	**F**	**25**	**-**	**-**	S
**277**	**6**	**5**	**0**	**6**	**F**	**32**	**-**	**-**	S
**37**	**0**	**0**	**8**	**8**	**M**	**49**	**-**	**-**	S
**117**	**8**	**5**	**2**	**8**	**F**	**46**	**-**	**-**	S
**317**	**0**	**0**	**9**	**9**	**F**	**38**	**-**	**-**	S
**397**	**9**	**2**	**N/A**	**9**	**M**	**26**	**-**	**-**	S

N/A, not available.

a Highest number of symptoms reported on either questionnaire.

b Female (F) and male (M).

c Antibody interpretation at index: positive (+), negative (−), or equivocal (E).

d AS is for asymptomatic when peak symptom number ≤3 and S is for symptomatic when peak symptom number ≥4.

**Figure 2:**
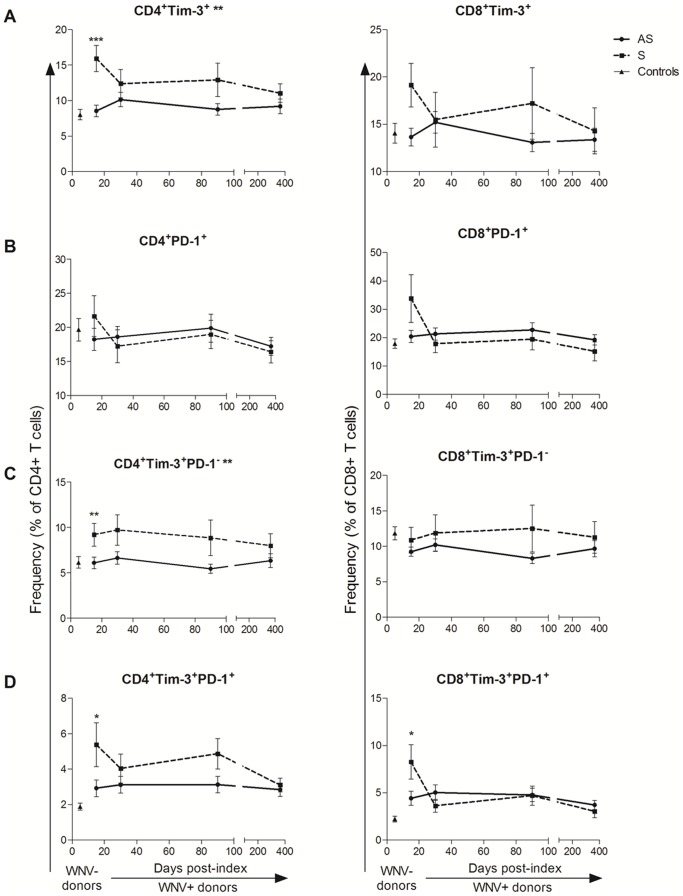
Frequencies of Tim-3 and PD-1 expressing T cells compared over the course of WNV infection in groups with different disease outcome. The graphs below demonstrate the change of frequencies of (A) Tim-3^+^, (B) PD-1^+^, (C) Tim-3^+^PD-1^−^, and (D) Tim-3^+^PD-1^+^ CD4^+^ (left panel) and CD8^+^ (right panel) T cell subsets in asymptomatic (AS, circles/solid lines, n = 24) and symptomatic (S, squares/dash lines, n = 8) WNV-infected subjects 14, 30, 90, and 365 days post-index donation. The frequencies of the same cells in uninfected controls are displayed (WNV−, triangles, n = 26). The symbols indicate the means and the error bars represent the SEM. The *p*-values of the pairwise comparisons between asymptomatic and symptomatic groups are indicated by *p <0.05, ** *p* <0.01, and *** *p* <0.001 above time-points when groups were compared at a given time-point by Mann-Whitney test. The *p*-values of the comparison between asymptomatic and symptomatic groups are indicated by ** adjacent to the cell subset title for *p* <0.01 over the time of post-index by generalized estimated equation (GEE).

WNV+ subjects could be segregated based on their clinical presentation during the acute viremic phase into asymptomatic and symptomatic WNV+ subjects (**Table 1**) [14]. We observed higher frequencies of total Tim-3^+^ CD4^+^ T cells in symptomatic WNV+ subjects compared to asymptomatic WNV+ subjects as early as day 14 post-index (*p* = 0.0009 FDR = 0.003) (**Figure 2A**) with symptomatic WNV+ subjects maintaining significantly higher frequencies of Tim-3^+^ CD4^+^ T cells throughout the course of WNV infection (overall GEE, *p* = 0.004) (**Figure 2A**). In contrast, asymptomatic and symptomatic WNV+ subjects expressed similar frequencies of PD-1^+^ T cells (**Figure 2B**). Symptomatic WNV+ subjects exhibited significantly higher frequencies of Tim-3^+^PD-1^−^ CD4^+^ T cells (*p*<0.05 FDR<0.05) (**Figure 2C**) and Tim-3^+^ PD-1^+^ CD4^+^ T cells (*p* = 0.015 FDR = 0.06) (**Figure 2D**) as well as Tim-3^+^ PD-1^+^ CD8^+^ T cells (*p* = 0.023 FDR = 0.09) than asymptomatic WNV+ subjects at day 14 post index.

### Symptomatic WNV+ subjects exhibit higher frequencies of early differentiated (CD28^+/−^CD57) Tim-3^+^ T cell populations

**Figure 3:**
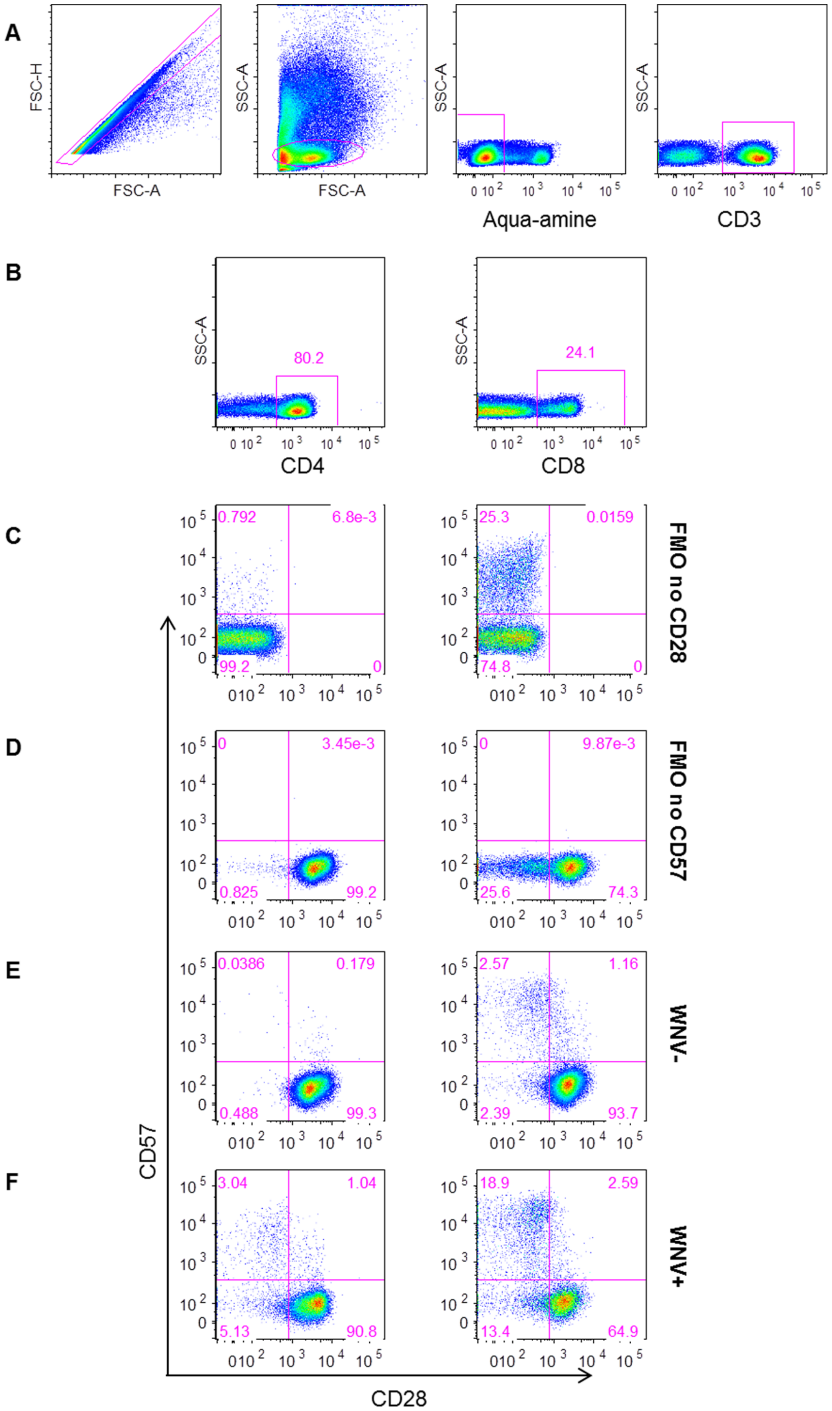
Gating strategy for measuring CD28 differentiation and CD57 senescence makers on T cells. The plots show (A) the gating strategy for live CD3^+^ lymphocytes, (B) for CD4^+^ (left) and CD8^+^ (right) T cells. Gates were set on FMO no CD28 (C) and FMO no CD57 (D). Plots are shown for representative (E) West Nile virus (WNV) uninfected controls (WNV−) and (F) WNV infected subjects (WNV+) day 14 post-index donation.

**Figure 4:**
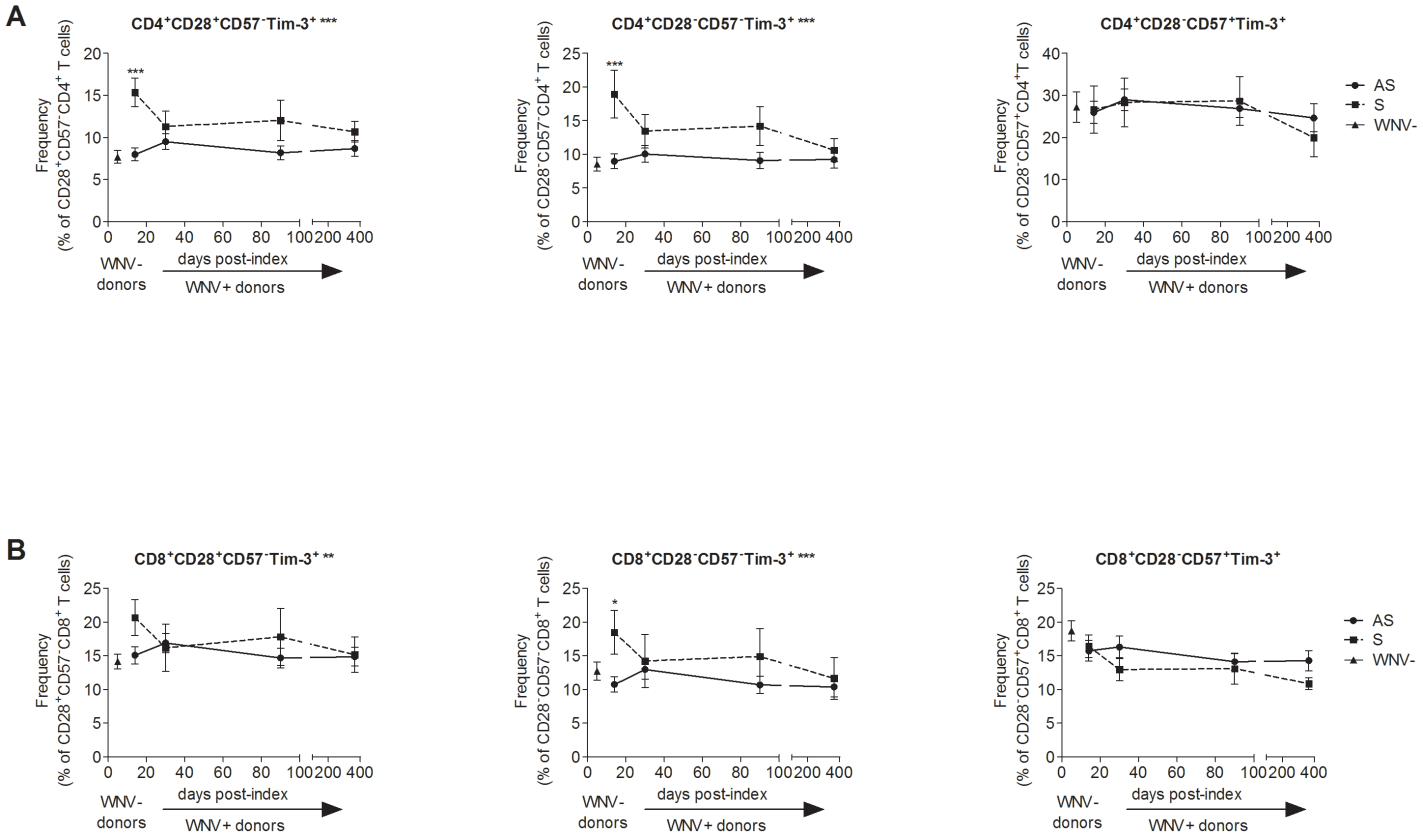
Differentiation status and functional capacity of Tim-3^+^ T cells in acute West Nile virus infection. The graphs show, through the course of WNV infection, the frequencies of Tim-3^+^ CD28^+^CD57^−^, CD28^−^CD57^−^ and CD28^−^CD57^+^ (A) CD4^+^ and (B) CD8^+^ T cell subsets in asymptomatic (AS, circles/solid lines, n = 24) and symptomatic (S, squares/dash lines, n = 8) WNV+ subjects 14, 30, 90, and 365 days post-index donation. The frequencies of the same cells in uninfected controls are displayed (WNV-, triangles, n = 26). The symbols indicate the means and the error bars represent the SEM. **p <0.01, *p <0.05, and *** *p* <0.001 by Mann-Whitney. The *p*-values of the comparison between asymptomatic and symptomatic groups are indicated by ** adjacent to the cell subset title for *p* <0.01 over the time post-index by GEE.

We measured the frequencies of differentiated T cells based on CD28 and CD57 expression over the course of WNV infection by flow cytometry (**Figure 3**) to assess the frequencies of Tim-3^+^ T cells within naïve/early differentiating (CD28^+/−^CD57^−^), differentiated (CD28^−^CD57^−^), and senescent (CD28^−^CD57^+^) T cell subsets in asymptomatic and symptomatic WNV+ subjects (**Figure 4**). Symptomatic WNV+ subjects had higher frequencies of Tim-3^+^ naïve/early differentiating and CD4^+^ T cells compared to asymptomatic WNV+ subjects from the earliest time-point at day 14 post-index (*p* AS vs. S<0.0001, FDR = 0.002) and throughout WNV infection (overall GEE, *p*<0.01 for both subsets) (**Figure 4A**). Symptomatic WNV+ subjects also exhibited higher frequencies of Tim-3^+^ differentiated CD8^+^ T cells than the asymptomatic WNV+ subjects at 14 days post-index (*p* AS vs. S = 0.02, FDR = 0.107) and throughout WNV infection (overall GEE, *p* = 0.043) (**Figure 4B**). Notably, asymptomatic and symptomatic WNV+ subjects expressed similar frequencies of senescent T cells throughout and after resolution of WNV infection.

### While Tim-3^+^ T cells are responsible for most of the IFN-γ secretion, both Tim-3^+^ and Tim-3^−^ T cells are competent for IFN-γ secretion

**Figure 5:**
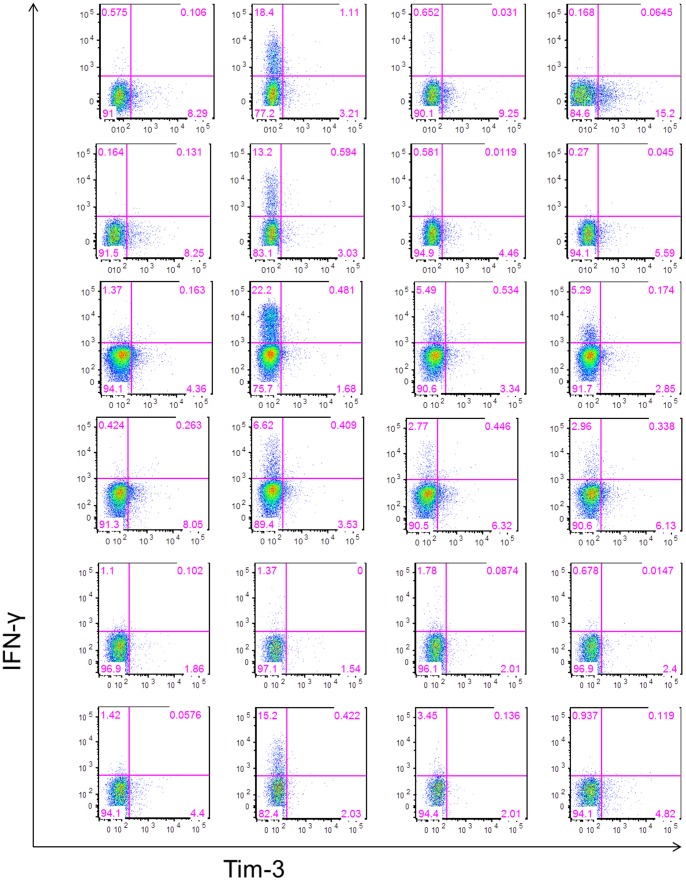
Gating strategy for phenotyping IFN-γ secreting T cells in response to stimulation. The frequencies of Tim-3^+^ and Tim-3^−^ IFN-γ secreting CD4^+^ and CD8^+^ T cells were measured in PBMC collected at day 30 post-index from 6 HLA-A02 WNV-infected donors and incubated with or without anti-CD3/anti-CD28 mAbs, WNV peptide pool, and SVG9 tetramer. Tim-3^−^ and Tim-3^+^ CD4^+^ and CD8^+^ T cells were analyzed for IFN-γ secretion. The gates were set using fluorescence minus one controls. Dot-plots for CD8^+^ T cells from all 6 WNV+ subjects in different stimulation conditions are shown.

**Figure 6:**
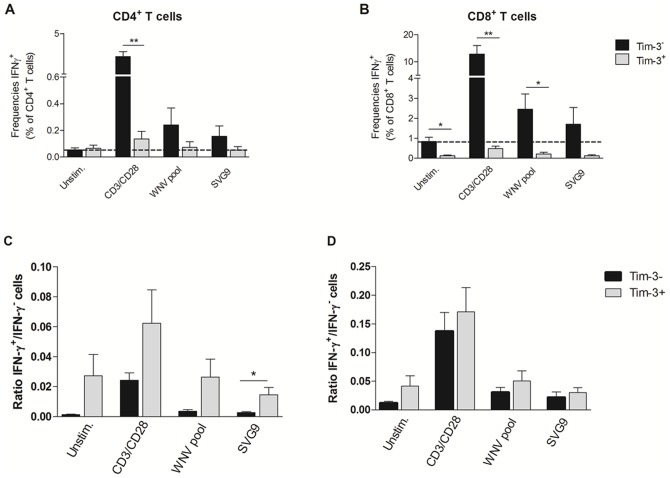
Phenotyping IFN-γ secreting T cells in response to stimulation. The frequency of IFN-γ-producing Tim-3^−^ and Tim-3^+^ CD4^+^ T cells (A) and CD8^+^ T cells (B) collected 30 days post-index from 6 HLA-A02 WNV-infected donors are shown after stimulation in the presence or absence of anti-CD3/anti-CD28 monoclonal antibodies, WNV peptide pool, and SVG9 peptide; Ratios of IFN-γ^+^/IFN-γ^−^ cells within Tim-3^−^ and Tim-3^+^ are shown for CD4^+^ T cells (C) and CD8^+^ T cells (D). The histograms indicate the means and the error bars represent the SEM. **p <0.01, *p <0.05, and *** *p* <0.001 by *t*-test.

To characterize further the functional state of the Tim-3^+^ T cells, we examined cytokine responses from PBMCs obtained from 6 HLA-A02 WNV+ donors incubated *ex vivo* with or without anti-CD3 and anti-CD28 monoclonal antibodies, a WNV peptide pool corresponding to immunodominant T cell epitopes in membrane, envelope, and nonstructural 3 and 4B proteins [17] **,** or an SVG9 peptide (HLA02 restricted SVGGVFTSV peptide in E) [16] by flow cytometry (**Figure 5**). In all conditions, the majority of IFN-γ-secreting T cells were Tim-3^−^ (**Figure 6A and B**). However, the ratios of IFN-γ^+^ cells within the Tim-3^−^ and Tim-3^+^ CD4^+^ (**Figure 6C**) and CD8^+^ T cells (**Figure 6D**) were similar if not higher in the Tim-3^+^ T cells.

## Discussion

In this longitudinal study, we assessed the dynamics of expression of immune inhibitory receptors during the evolution of acute WNV infection. Our experiments revealed that symptomatic WNV+ donors exhibited higher frequencies of Tim-3^+^ and Tim-3^+^PD-1^+^ T cells compared to asymptomatic WNV+ donors throughout the course of infection, and Tim-3^+^ populations phenotypically were CD28^+^ CD57^−^ naïve/differentiating and CD28^−^ CD57^−^ differentiated CD4^+^ and CD8^+^ T cells. This is the first report documenting the induction of Tim-3^+^PD-1^+^ T cells after WNV infection and the association between the frequency of Tim-3^+^ T cells and WNV disease outcome.

The increased expression of negative inhibitory receptors on T cells has been observed in chronic viral infections and cancers. Under these conditions, a phenomenon of T cell exhaustion develops that has been associated with higher antigen load or longer antigen exposure. Antibody blockade of exhaustion markers leads to an improvement in antiviral and anti-tumor T cell immunity, reduced viremia or tumor burden, and reduced severity of disease outcome [19], [20], [21], [22], [23]. Tim-3^+^PD-1^+^ T cell induction in the acute phase of HCV infection correlated with defective T cell responses and may contribute to the lack of immune control and development of persistence [19]. Unexpectedly, a recent study examining acute *Mycobacterium tuberculosis* infection in humans found that Tim-3^+^ T cells were more active and effective in suppressing mycobacterial infection in macrophages, suggesting a possible differential role for Tim-3 in an acute bacterial infection [24].

WNV infection is an acute viral infection, which in the majority of individuals resolves within a few weeks of exposure, although the virus may persist in some humans [25], [26] and mice [27]. The expression of T cell exhaustion markers, such as Tim-3 and PD-1, has not been investigated extensively in acute viral infections. One recent study, reported a protective role of Tim-3 in acute influenza infection in mice [28]. Our findings, in contrast, suggest that individuals with higher frequencies of Tim-3^+^ T cells after WNV infection experience symptomatic disease. In mice, severe disease following influenza infection may be due more to the immune response and less to the virus directly [29], whereas WNV directly promotes injury and death of some neuronal subtypes once it reaches the CNS [7]. Thus, the difference in pathogenesis and mechanism of injury may explain the discrepant results between acute influenza and WNV infection.

The reasons for increased frequencies of Tim-3^+^ T cells in symptomatic WNV+ subjects are not clear. Whether symptomatic WNV+ subjects developed higher frequencies of Tim-3^+^ T cells after WNV infection or had higher levels of these cells prior to infection remains to be established. With no pre-WNV infection samples available, our human studies are limited to an association between elevated Tim-3^+^ T cell frequencies and symptomatic disease. The functional characterization of T cells *ex vivo* after restimulation revealed that while Tim-3^+^ T cells secrete IFN-γ, most of the IFN-γ is secreted by Tim-3^−^ T cells. In the context of WNV infection, Tim-3^+^ T cells might be less functional, as reported in other infectious models [12]. Taken together, these results demonstrated that Tim-3^+^ T cells triggered after WNV infection *a priori* are not less functional in terms of IFN-γ secretion than the Tim-3^−^ T cells even though most of the IFN-γ response comes from Tim-3^−^ cells.

We observed an association between symptomatic disease outcome and Tim-3^+^ T cells with a CD28^+/−^CD57^−^ naïve/early differentiated cellular profile. Recent studies reported that Tim-3 expression can be induced in naïve [30], [31], effector, and memory T cells in an antigen-independent manner by cytokines, such as IL-2, IL-7, IL-15 and IL-21, and also can be expressed on the surface of naïve, effector, and memory T cells [30]. In the context of an acute viral infection this may impact the generation of an effective antiviral T cell response. We envision that during acute WNV infection, premature immune exhaustion among early differentiating T cells could blunt the T cell responses that are required to control and clear the virus, especially in the later phases of pathogenesis. Whether symptomatic WNV+ subjects develop higher, more persistent viremia and/or inflammation remains to be investigated. Placing our data into the context of prior studies showing that PBMCs from symptomatic WNV+ subjects’ PBMCs preferentially secrete IFN-γ in response to WNV peptide restimulation [17], and that lower levels of Tregs circulate in symptomatic WNV+ subjects [14], we propose that, in symptomatic WNV+ subjects, higher antigen load and inflammatory responses could lead to increased cellular activation, with Tim-3 marking a population of hyperactivated cells. Alternatively, sustained or high levels of viral infection and inflammation could induce expression of inhibitory receptors, with Tim-3^+^ on T cells acting to limit collateral immune-mediated damage to host tissues. Nonetheless, this counter-regulatory activity could be detrimental if blunted T cell responses allowed WNV to persist longer in the CNS, where the virus is cytopathic for neurons [7]. In this scenario, targeting of Tim-3^+^ T cells (antibody blockade or depletion) might boost the T cell immune response to control viral replication and reduce disease severity, as reported for chronic viral infections (HIV, HCV, and HBV) [13], [19], [32]. Clearly, further investigation is required to determine whether higher frequencies of Tim-3^+^ T cells are the cause or the consequence of symptomatic WNV disease.
